# Supplementary material for: Complex electric double layers in charged topological colloids
Source: Sci Rep. 2018 Sep 20;8:14119. doi: 10.1038/s41598-018-32550-8 (PMC6147863; doi:10.1038/s41598-018-32550-8)
Supplement: Supplementary file 1 — Supplementary information [file 41598_2018_32550_MOESM1_ESM.pdf]

Supplementary material: Complex electric double layers in charged topological colloids

J. C. Everts[1,*] and M. Ravnik[1,2]

[1]*Department of Physics, Faculty of Mathematics and Physics, University of Ljubljana, Jadranska 19, 1000 Ljubljana, Slovenia*

[2]*Department of Condensed Matter Physics, Jozef Stefan Institute, Jamova 39, 1000 Ljubljana, Slovenia*

(Dated: August 7, 2018)

* jeffrey.everts@gmail.com

Constant-charge boundary conditions In this paper we focussed on constant-potential boundary conditions, see Eq. (2) main text, which resulted in an inhomogeneous surface charge distribution on the particle. If we would instead fix the surface charge density σ to be spatially constant, however, the surface potential is inhomogeneous. In this case the constant-charge boundary condition reads

$$\mathbf{n} \cdot \left(\frac{\epsilon_p}{\epsilon_s} \right) \nabla \phi(\mathbf{r})|_{\text{in}} - \mathbf{n} \cdot \nabla \phi(\mathbf{r})|_{\text{out}} = 4\pi \lambda_B \sigma, \quad \mathbf{r} \in \Gamma,$$

with ϵ_p the dielectric constant of the particle and ϵ_s the dielectric constant of the surrounding solvent. The exact ratio ϵ_p/ϵ_s only affects the surface potential by a few percent, so we fixed it at $\epsilon_p = 2.6$ (PMMA) and $\epsilon_s = 80$ (water), although the exact values are irrelevant within the numerical accuracy that we are interested in. Note, moreover, that for constant-potential boundary conditions the inner gradient vanishes, $\nabla \phi|_{\text{in}} = 0$.

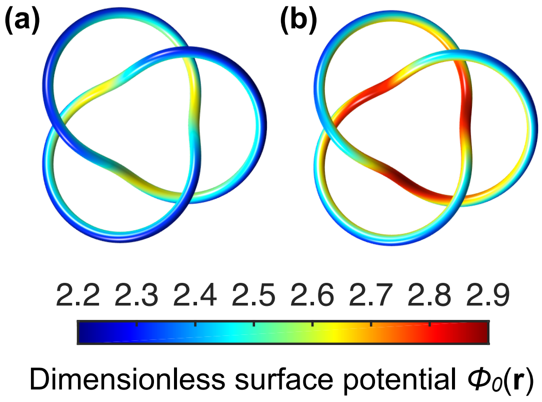

Figure S1: Surface potential distribution $\Phi_0(\mathbf{r})$ for constant-charge boundary conditions. We use the same parameters as in Figure 3 for a trefoil knot particle, however with a fixed surface charge density σ that coincides with the maximal surface charge density σ_{max} from Figure 3(a) and (b), respectively. Specifically, in (a) we have $\sigma a \lambda_B = 0.083$ and Debye length $\lambda_D = 7.4a$, with a the tube radius and λ_B the Bjerrum length. In (b) we have $\sigma a \lambda_B = 0.068$ and $\lambda_D = 11.6a$.

We demonstrate the constant-charge boundary conditions for a trefoil knot particle where we choose σ such that it coincides with the maximal charge density σ_{max} of the constant-potential particle in Figure 3. In Figure S1 we see that the resulting surface potential distribution $\Phi_0(\mathbf{r})$ is lowest at the outer rim of the particle tube and close to particle-tube crossings. This is in line with results for spherical particles where it is known that constant-charge particles at low volume fraction have a lower surface potential than at high volume

fractions [1].

Finally, we observed that the topological transformation is exactly the same as in Figure 3 (not shown since isosurfaces are identical as in Figure 3(e)), albeit the transition between different topological shapes occur at slightly different Debye lengths. The only difference with the constant-potential result is therefore that isosurfaces of the screening cloud close to the particle surface might not enclose the whole particle, which is an immediate result of the inhomogenous surface-potential distribution $\Phi_0(\mathbf{r})$.

Screening cloud isosurfaces In Supplementary movie 1 we show the isosurfaces of Figure 3(e) in the main text from various camera angles.

Surface charge density of torus knots In Figure S2 we show the charging behaviour of the torus knots discussed in the main text in Figure 4.

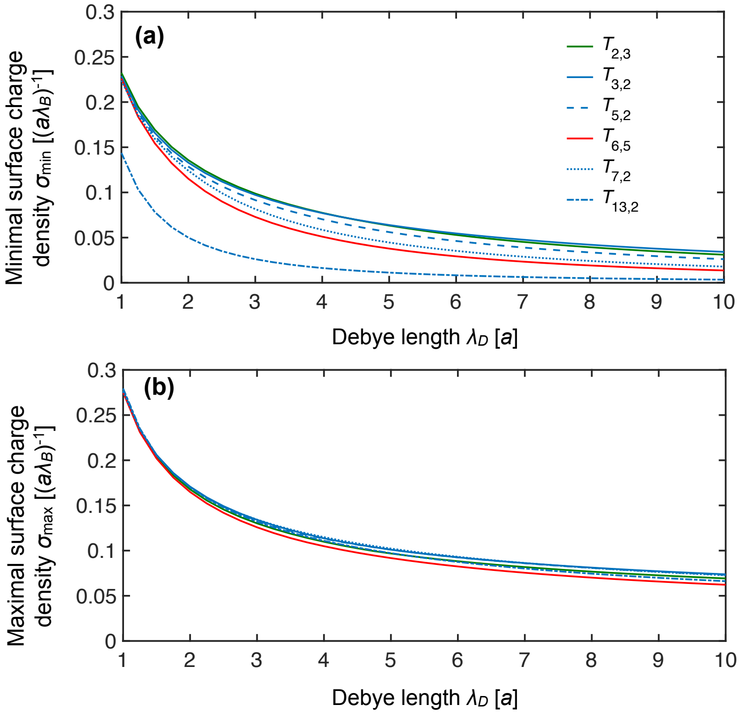

Figure S2: (a) Minimal and (b) maximal values of the surface charge density $\sigma_{\min}$ and $\sigma_{\max}$, respectively for various torus knots $T_{p,q}$ for a range of Debye lengths λ_D with the same parameters as in Fig. 4. Here a is the tube radius of the torus knot, and λ_B is the Bjerrum length of the medium.

[1] Everts, J. C., Boon, N. & van Roij, R. Density-induced reentrant melting of colloidal wigner crystals. *Phys. Chem. Chem. Phys.* **18**, 5211–5218 (2016).
